# RBC Membrane‐Camouflaged Nanosystem‐Mediated Synergistic Drug Combination for Enhanced Anti‐Tumor Therapy

**DOI:** 10.1002/adhm.202500446

**Published:** 2025-05-07

**Authors:** Qian Cheng, Xuemei Zhong, Lu Deng, Xinling He, Miaoxizi Luo, Ruibing Wang, Jinming Zhang

**Affiliations:** ^1^ State Key Laboratory of Southwestern Chinese Medicine Resources Pharmacy School Chengdu University of Traditional Chinese Medicine Chengdu 611130 China; ^2^ State Key Laboratory of Quality Research in Chinese Medicine Institute of Chinese Medical Sciences University of Macau Taipa Macau SAR 999078 China

**Keywords:** anti‐tumor, CPPs, drug combination, nanosystem, RBC membrane‐camouflaged

## Abstract

Achieving direct delivery of drugs to the tumor site is a prerequisite for successful drug development, necessitating that drug molecules be cell permeable. Numerous studies have shown that cell‐penetrating peptides (CPPs) can augment the efficacy of various therapeutic agents by enhancing cellular uptake, prompting their advancement into preclinical studies. However, the non‐specificity and cationic properties of CPPs impede their clinical application. Attributed to the easily accessible capacity and excellent biocompatibility of red blood cells (RBC), herein, an RBC membrane camouflaged, cell‐penetrating peptides R8‐(RRRRRRRR)based drug delivery system is constructed to achieve a synergistic combination of natural compounds triptolide (TP) and celastrol (Cel), thus precisely inducing tumor inhibition. The RBC membrane camouflaged nanosystem escaped from the reticuloendothelial system (RES) and is endocytosed by tumor cells mediated by R8. Released TP and Cel‐induced tumor cell apoptosis, reducing tumor metastasis invasion and triggering autophagy disorder in breast cancer and liver cancer. Overall, the biomimetic nanosystem realizes enhanced drug combination therapy with high‐level biosafety, which provides a facile strategy to improve the clinical application of CPPs‐based drug combinations.

## Graphical abstract

An RBC membrane camouflaged, cell‐penetrating peptides R8‐based drug delivery system is constructed to achieve a synergistic combination of natural compounds triptolide (TP) and celastrol (Cel), inducing tumor cell apoptosis, reducing tumor metastasis invasion and triggering autophagy disorder in breast cancer and liver cancer.

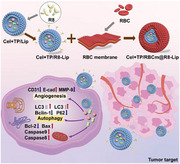

## 1 Introduction

Malignant tumors remain one kind of the deadliest diseases worldwide.^[^ ^1^ ^]^ Currently, chemotherapy remains a primary modality for treating malignant tumors compared with surgery and radiation therapy, which can only exert local therapeutic effects. The effective delivery of chemotherapeutic agents to tumor sites is a focal point of numerous studies. The development of targeting strategies holds promise for enhanced specific drug delivery to tumor cells.^[^ ^2^ ^]^ Existing technologies typically rely on passive or active targeting to transport payloads to specific sites, thereby mitigating systemic side effects and augmenting therapeutic outcomes. Active biological recognition by antibodies or other biomolecules to direct drugs to specific intracellular sites is often employed to enhance targeting efficiency,^[^ ^3^ ^]^ necessitating that drug molecules be cell‐permeable. The traversal of the plasma membrane presents a challenge for certain therapeutic agents, as numerous drugs exhibit optimal activity in vitro yet demonstrate significantly reduced activity in vivo.^[^ ^4^ ^]^ Consequently, optimizing the cellular delivery of therapeutic agents is of paramount importance. Numerous studies have shown that CPPs, for example, R8 (RRRRRRRR), can augment the efficacy of various therapeutic agents by enhancing cellular uptake, prompting their advancement into preclinical studies.^[^ ^5^ ^]^ However, CPPs generally exhibit a lack of tissue selectivity, capable of penetrating all cellular membranes.^[^ ^6^ ^]^ Furthermore, due to their positive charge, R8 binds to plasma proteins during circulation, thereby reducing their in vivo stability. These limitations significantly hinder the clinical application of CPPs.^[^ ^7^ ^]^ Therefore, there is an urgent need to develop biocompatible drug delivery strategies to solve the problem of clinical conversion.

Biomimetic cell membrane‐camouflaged nanoparticles have emerged as the next generation of particle‐based therapeutics, with increasingly widespread applications while imparting the various biological functions of cellular surfaces to the nanoparticles.^[^ ^8^ ^]^ Typically, these biomimetic nanoparticles are formed by coating nanoparticles with cell membranes extracted and isolated from red blood cells (RBCs), platelets, cancer cells, mesenchymal stem cells, or white blood cells, creating a core/shell structure. Among them, RBC membranes, due to their natural transport role in blood circulation, emerge as excellent membrane‐biomimetic materials, exhibiting favorable biocompatibility, low immunogenicity, and extended circulation properties.^[^ ^9^ ^]^ For instance, it has been reported that coating PLGA nanoparticles with RBC membranes can prolong blood circulation time and reduce macrophage phagocytosis,^[^ ^10^ ^]^ attributed to the “self‐marker” protein CD47 expressed on the membrane, which inhibits immune responses.^[^ ^11^ ^]^Additionally, the deformability of RBCs enhances drug penetration, facilitating accumulation within tumor tissues.^[^ ^12^ ^]^

Thus, in this work, a liposome drug delivery system based on transmembrane peptide R8 and RBC membrane camouflage strategy was designed. As proof of concept, natural compounds triptolide (TP) and celastrol (Cel), as potential natural anticancer agents, were loaded into the liposome. As two main components of Tripterygium wilfordii, few studies have combined TP and Cel to investigate their synergistic effect. Limited reports also use free drugs, but the side effects caused by drug solubility and targeting seriously hinder the application of both TP and Cel. Specifically, A combination of TP and Cel can enhance efficacy with synergistic effects, from which, TP can exert anti‐tumor effects through various pathways such as induction of apoptosis, cycle arrest, antioxidant, inhibition of invasion and metastasis, induction of autophagy, and mediation of cancer cell immunity.^[^ ^13^ ^]^ Its homologous component, Cel, a proteasome inhibitor, can inhibit proteasome activity in tumor cells, accumulate proteasome target proteins induce apoptosis, and has a good inhibitory effect on many kinds of tumor cells.^[^ ^14^ ^]^ This TP/Cel biomimetic liposomes design has several potential advantages. Primarily, both TP and Cel can be fully encapsulated into liposomes and simultaneously transported to the tumor site with a synergistic ratio. In the circulation, the RBC membranes mimetic liposomes maintain stability and effectively shield the transmembrane peptide R8. Upon arrival at the tumor site, R8 is exposed, concurrently facilitating the internalization of the liposomes into tumor cells. This study presents a drug delivery system featuring liposomes co‐decorated with RBC membranes and transmembrane peptides. It examines the influence of surface proteins on cellular uptake capacity and immune evasion. Additionally, the in vivo targeting efficacy, pharmacodynamics, and underlying mechanisms of this system are assessed in both breast cancer and liver cancer models.

## 2 Results and Discussion

### 2.1 Synergistic Effect of TP and Cel

First, the synergistic effect of TP and Cel was evaluated against HepG2 and MCF‐7 cancer cells. As shown in Figure S1 (Supporting Information), TP/Cel mixture induced significant effects on cell proliferation on both HepG2 and MCF‐7 cell lines at a TP to Cel molar ratio of 1:100 with various concentrations in a synergistic manner when compared to the free TP and free Cel group.^[^ ^15^ ^]^ The IC50 values of TP/Cel mixture were 1028±8.501 nm on HepG2 cell and 1695±7.496 nm on MCF7 cell, respectively. While TP exhibited minimal effects in the range of concentrations we used. And the IC50 values of Cel on HepG2 and MCF‐7 cells were 1223 ± 7.491 and 2099 ± 7.081 nm, respectively. These results suggested that the mixture of TP and Cel may enable an efficient synergy effect suitable for cancer therapy in the combination therapy.

### 2.2 Preparation and Characterization of Cel+TP/RBCm@R8‐Lip

**Scheme 1:**
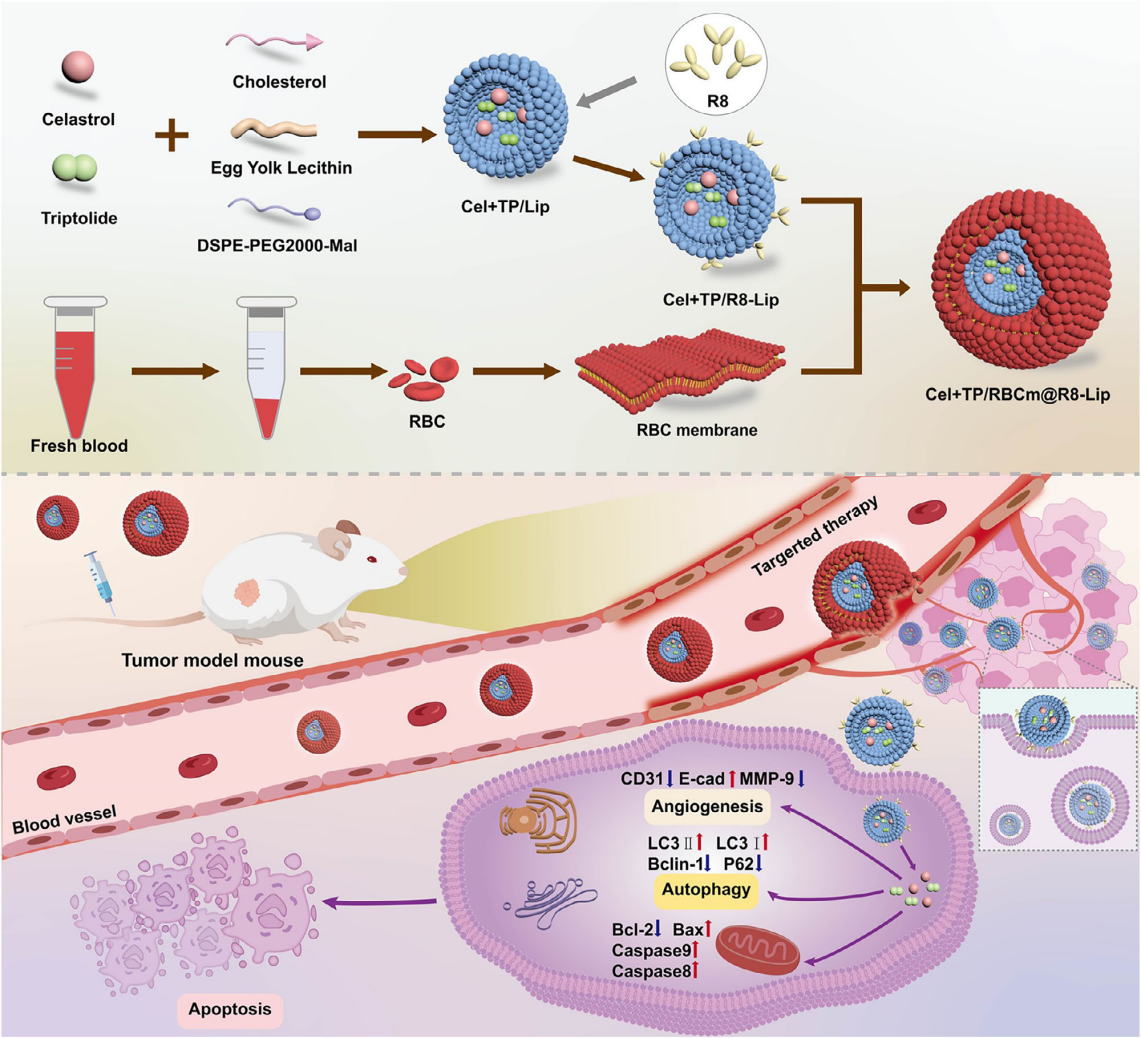
The preparation process of Cel+TP/RBCm@R8‐Lip and synergistic therapy of TP and Cel co‐loaded in RBC membranes‐camouflaged and transmembrane peptide R8‐based liposomes.

**Figure 1:**
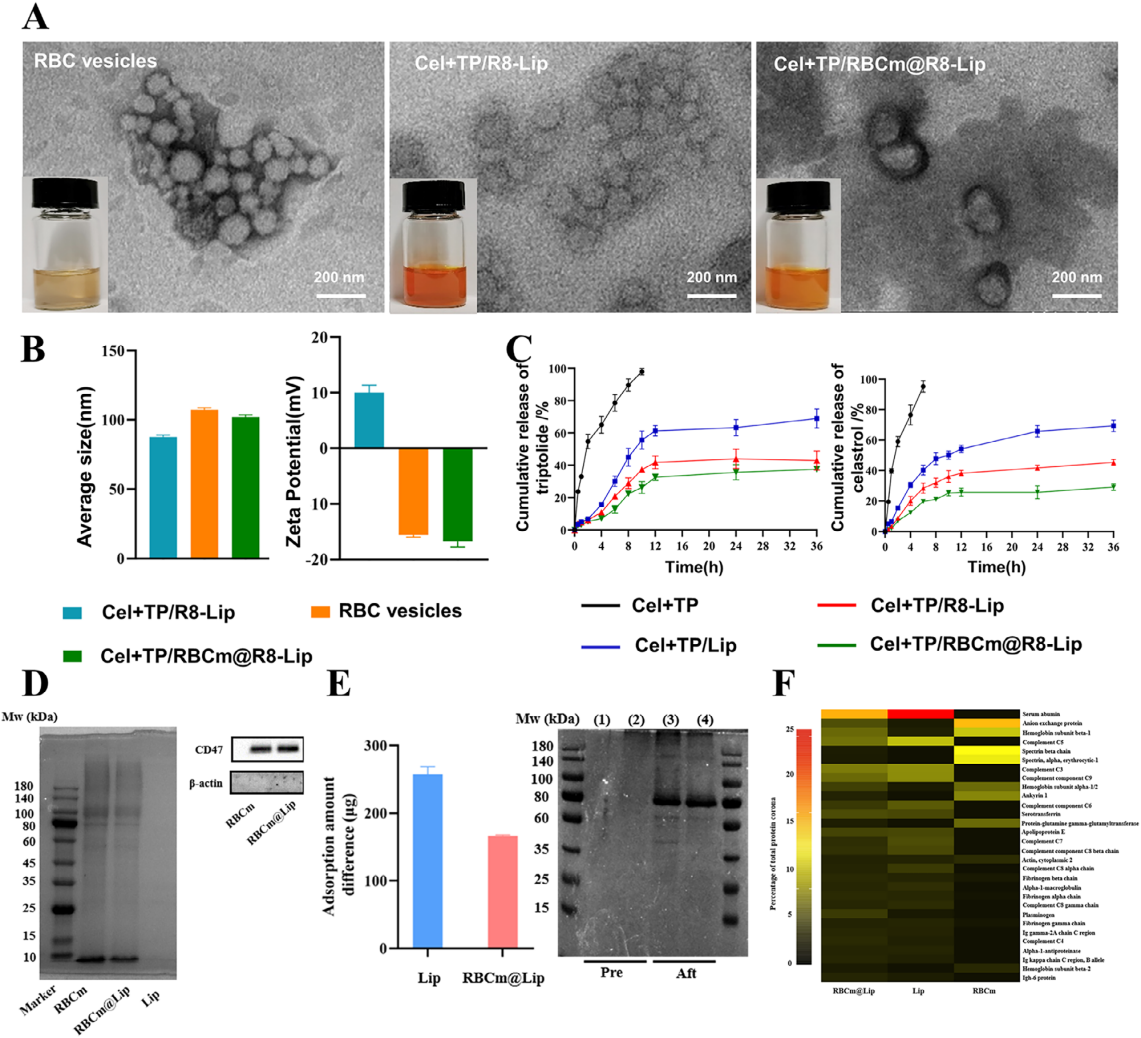
Characterization of Cel+TP/RBCm@R8‐Lip and proteins corona analysis. A) Representative photos and TEM images of aqueous suspension of RBC vesicles, Cel+TP/R8‐Lip, Cel+TP/RBCm@R8‐Lip. B) Particle sizes and zeta potentials of RBC vesicles, Cel+TP/R8‐Lip and Cel+TP/RBCm@R8‐Lip analyzed by DLS. C) Drug release profiles of triptolide and celastrol (n = 3). D) Characteristic protein bands of RBC vesicles, Lip, RBCm@Lip resolved by SDS‐PAGE and Western blotting. E) Quantitative and qualitative analysis of protein changes after Lip and RBCm@Lip co‐incubation with serum, respectively. 1) Lip before co‐incubation. 2) RBCm@Lip before co‐incubation. 3) Lip after co‐incubation and (4) RBCm@Lip after co‐incubation. F) Heat map of the most abundant proteins in the protein corona. Proteins that constitute at least 1% of the protein corona on the liposomes are shown. All data are presented as mean ± SD.

Next, the Cel and TP were encapsulated into the R8 decorated liposomes (Cel+TP/R8‐Lip) using ethanol injection method. Then the RBC vesicles were collected for the surface modification. Cel+TP/RBCm@R8‐Lip was prepared by fusing Cel+TP/R8‐Lip with RBC membrane via sonication (**Scheme** **1**). The Transmission Electron Microscope (TEM) images showed the morphology of Cel+TP/RBCm@R8‐Lip was typically spherical, and the inner and outer layers were clearly visible (**Figure** **1**A). The Dynamic Light Scattering (DLS) analysis indicated excellent particle size distribution of RBC vesicles and Cel+TP/RBCm@R8‐Lip. Moreover, the hydrodynamic diameters of Cel+TP/R8‐Lip and Cel+TP/RBCm@R8‐Lip were 88.69 nm with a Polymer Dispersity Index (PDI) of 0.205 and 99.61 nm with a PDI of 0.201, respectively. The zeta potential analysis of Cel+TP/RBCm@R8‐Lip showed similarly negative surface charge of RBC vesicles (Figure 1B), indicating the successful membrane coating of Cel+TP/RBCm@R8‐Lip.^[^ ^16^ ^]^ The encapsulation efficiency (EE) of TP and Cel was 86.3 ± 1.63% and 95.6±1.33%, respectively. Next, the in vitro drug release profiles of TP and Cel from Cel+TP, Cel+TP/Lip, Cel+TP/R8‐Lip, and Cel+TP/RBCm@R8‐Lip in PBS were examined. As illustrated as Figure 1C, during the first 6 h, 40.32%, 28.46%, and 19.76% of Cel were released from Cel+TP/Lip, Cel+TP/R8‐Lip and Cel+TP/RBCm@R8‐Lip, respectively. At 10 h, 55.6%, 37.4%, and 26.29% of TP were released from Cel+TP/Lip, Cel+TP/R8‐Lip, and Cel+TP/RBCm@R8‐Lip, respectively, indicating that all three liposomes exhibited sustained drug release profiles. However, due to the dual‐coating of R8 and RBC vesicles, Cel+TP/RBCm@R8‐Lip exhibited comparatively slower drug release profiles than that of Cel+TP/Lip during the 36 h release, the cumulative release rate of TP and Cel from Cel+TP/RBCm@R8‐Lip at final 36 h was 37.56% and 29.15%.

### 2.3 Surface Characterization and Protein Corona Analysis

RBC membranes of endogenous molecules can elicit prolonged blood circulation and immune evasion. To verify the inheritance of membrane proteins on RBCm@R8‐Lip derived from red blood cells (RBCs), the protein profile of these nanocarriers was investigated through Sodium Dodecyl Sulfate Polyacrylamide Gel Electrophoresis (SDS‐PAGE) analysis. Figure 1D exhibited that the protein profiles of RBCm@Lip were identical to those of RBC membranes. Furthermore, the disappearance of β‐actin bands in analysis of the Western Blot (WB) test indicated that during the extraction process of erythrocyte membranes, the erythrocytes were adequately ruptured, leading to the complete release of their contents. Additionally, the retention of the key membrane antigen CD47, which blocks the phagocytosis of macrophages, was confirmed on the surfaces of both RBC membranes and RBCm@Lip, indicating the successful modification of the RBC membrane onto the liposomes, maintaining the biological functions of RBC membranes, thereby developing covert effects for potential drug delivery.

Since, upon entering the blood circulation, the nanoparticles will encounter various components including enzymes, plasma proteins, the reticuloendothelial system (RES), phagocytes, and others, resulting in the formation of a protein crown, a protein layer, on their surface.^[^ ^17^ ^]^ These protein‐coated nanoparticles are readily recognized by the immune system, subsequently leading to rapid clearance by the mononuclear phagocyte system (MPS).^[^ ^18^ ^]^ To investigate whether coating with RBC membranes can mitigate the formation of protein crowns on RBCm@Lip, the protein content, composition, and category of the protein crown were analyzed using a Bicinchoninic Acid Assay (BCA) kit, SDS‐PAGE, and Liquid Chromatograph Mass Spectrometer/Mass Spectrometry (LC‐MS/MS), respectively. Notably, as depicted in Figure 1E, it is noteworthy that fewer proteins adsorbed by the RBCm@Lip compared with pure liposomes after incubating with plasma for 0.5 h. Further analysis of the specific protein types within the protein crown (PC) by LC–MS/MS, a heatmap of the proteins contained in the protein coronas showed that the amount of immune response opsonins such as immunoglobulins, fibrinogens, and complement proteins adsorbed on the the RBCm@Lip was markedly reduced compared to Lip group (Figure 1F). Therefore, through the RBC membrane camouflage strategy, the minimized immunoglobulin adsorption within the protein corona of RBCm@Lip prevented interaction with complement proteins and may exhibit lower immunogenicity in vivo.

### 2.4 Cellular Uptake and In Vitro Cytotoxicity

**Figure 2:**
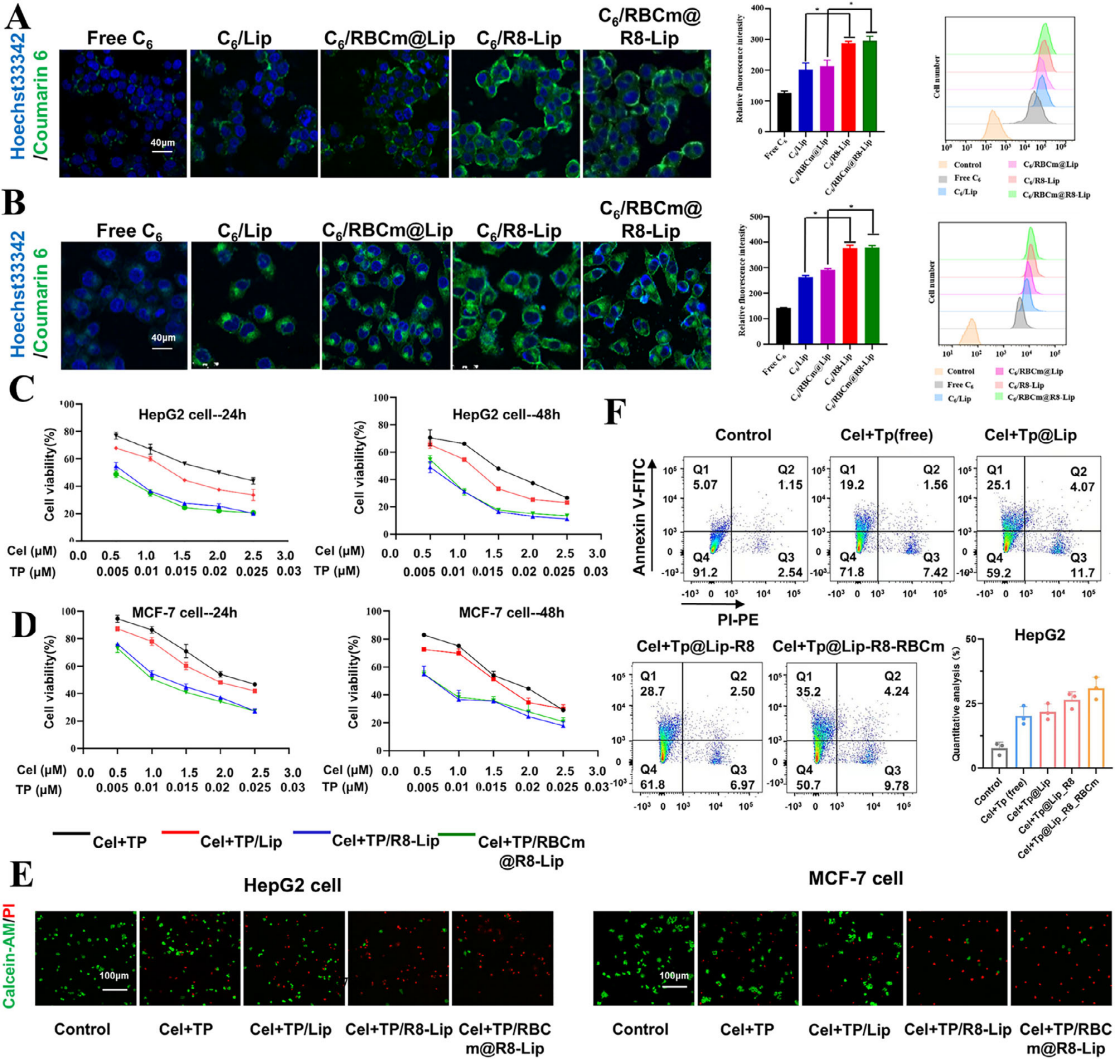
Cellular uptake and in vitro cytotoxicity. Representative cellular uptake images of A) HepG2 and B) MCF‐7 cells after incubation with Free C6, C6/Lip, C6/RBCm@Lip, C6/R8‐Lip, C6/RBCm@R8‐Lip, respectively, for 4 h by CLSM observation and by FCM. Scale bar: 40 µm (n = 3). And Cytotoxicity of Free Cel+TP, Cel+TP/Lip, Cel+TP/R8‐Lip, Cel+TP/RBCm@R8‐Lip against C) HepG2 and D) MCF‐7 cells with 24 h and 48 h treatment in different culture media. E) Detection of live/dead cells after various therapies. Scale bar: 100 µm. F) Qualitative and quantitative of AnnexinV‐FITC and PI‐PE staining of HepG2 cells treated with Free Cel+TP, Cel+TP/Lip, Cel+TP/R8‐Lip, Cel+TP/RBCm@R8‐Lip for 24 h (n = 3). All data are presented as mean ± SD. One‐way ANOVA with Tukey's multiple comparison test was used for statistical analysis. ^*^ *p* < 0.05.

The current drug delivery systems often face rapid clearance by Mononuclear Phagocyte System (MPS) before reaching tumor site.^[^ ^19^, ^20^ ^]^ Therefore, the macrophage (RAW 264.7) was employed to investigate the cellular uptake behavior of RBCm@R8‐Lip qualitatively and quantitatively by confocal laser scanning microscopy (CLSM) and flow cytometry (FCM). In order to better track the fluorescence of liposomes in cells, C6, a green fluorescence dye, was encapsulated into liposomes as a fluorescent tracker. As shown in Figure S2A (Supporting Information), weak fluorescence was observed in macrophage after incubation of C6/RBCm@R8‐Lip and C6/RBCm@Lip for 4 h. In comparison, the C6/Lip and C6/R8‐Lip groups showed strong fluorescence, indicating that the liposomes camouflaged by RBC membranes may provide an effective strategy to escape from the clearance of MPS, benefiting the prolonged circulation in vivo. Once accumulated in the tumor site, the R8 will enhance the drug uptake behavior of tumor cells. Thus, cellular uptake behaviors of tumor cells, HepG2 and MCF‐7 cells, were conducted by CLSM and FCM analysis. As shown in **Figure** **2**A,B and S5 (Supporting Information), CLSM images revealed the rapid cellular uptake of both R8‐modified liposomes C6/R8‐Lip and C6/RBCm@R8‐Lip with strong green fluorescence observed in the HepG2 and MCF‐7 cells after incubation for 4 h. In contrast, C6/Lip and C6/RBCm@Lip treated tumor cells had relatively weaker fluorescence. Quantification results measured by flow cytometry also confirmed that R8‐modified liposomes possessed faster cellular uptake behaviors by HepG2 and MCF‐7 cells owing to the enhanced cell uptake effect of cell‐permeating peptide R8.

Subsequently, chemotherapeutic drugs Cel and TP were encapsulated into these liposomes. The in vitro cytotoxicity of Cel+TP, Cel+TP/Lip, Cel+TP/R8‐Lip, and Cel+TP/RBCm@R8‐Lip were evaluated against HepG2 and MCF‐7 tumor cells using Cell Counting Kit‐8 (CCK‐8) assay. As shown in Figure 2C,D, Cel+TP/R8‐Lip group and Cel+TP/RBCm@R8‐Lip group exhibited dose‐dependent toxicity against HepG2 and MCF‐7 tumor cells, which were more toxic than other groups. Furthermore, live/dead cell staining was also conducted, as shown in Figures 2E and S6 (Supporting Information), both Cel+TP/R8‐Lip group and Cel+TP/RBCm@R8‐Lip group exhibited intense red fluorescence indicating the strong cell growth inhibition effect, which is consistent with CCK‐8 analysis results. We further evaluated whether Cel+TP/RBCm@R8‐Lip would enhance apoptosis in tumor cells. The apoptosis experiment was analyzed via annexin V‐fluorescein isothiocyanate (FITC)/propidium iodide‐phycoerythrin (PI‐PE) staining. As shown in Figure 2F, after incubation with the equivalent concentration of 0.01 µm TP and 1 µM Cel for 24 h, all liposome formulations produced a higher rate of apoptosis than that of free TP and Cel. Especially, Cel+TP/RBCm@R8‐Lip induced the highest apoptotic rate, contributing to its better cellular uptake behaviors.

### 2.5 In Vivo Biodistribution and Antitumor Efficacy Against H22 Tumor‐Bearing Mice

**Figure 3:**
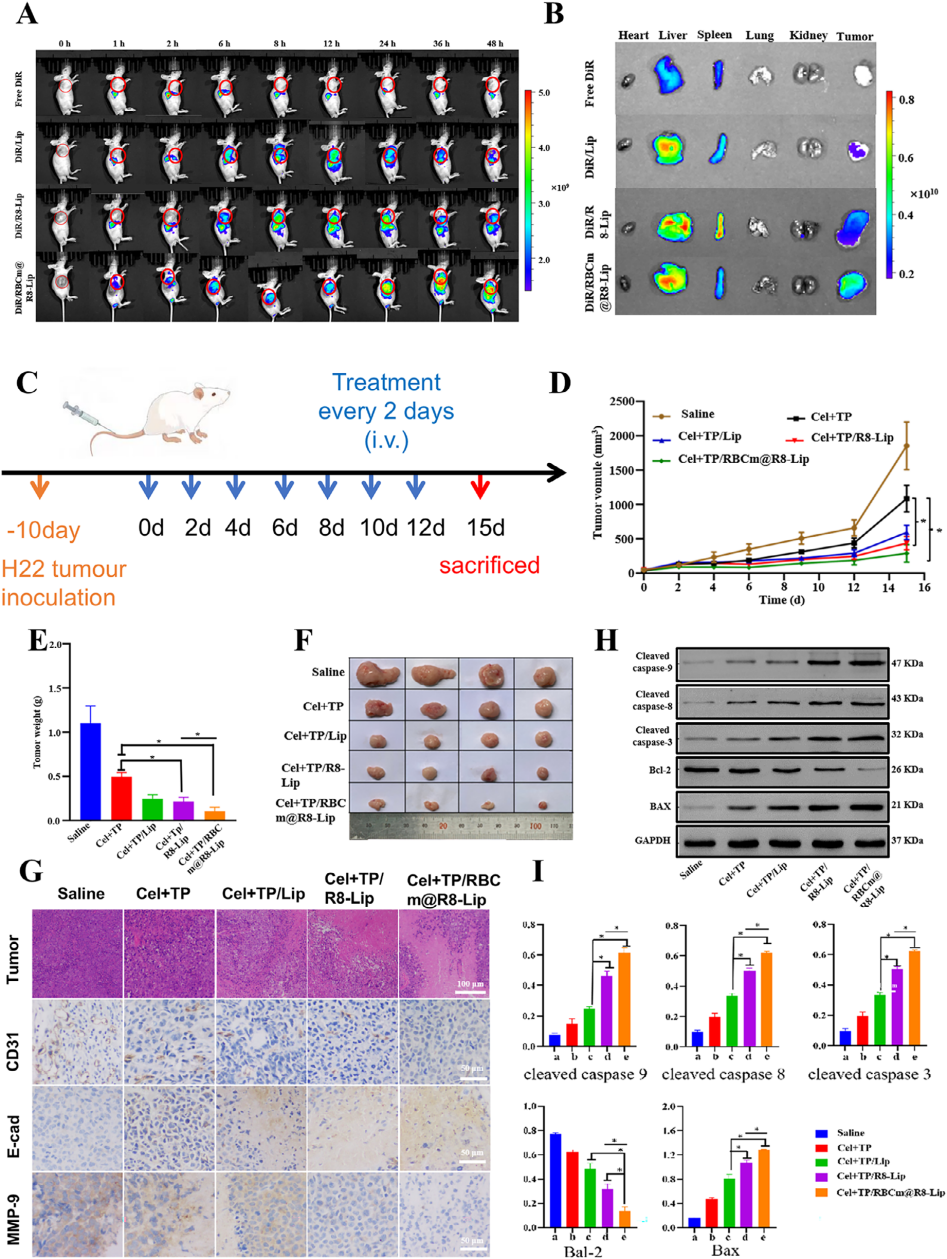
In vivo biodistribution and anti‐tumor effects of biomimetic liposomes in H22‐bearing tumor mice model. A) H22 tumor‐bearing mice's fluorescent distribution after vein injection at predetermined time intervals, i.e., 0, 1, 2, 6, 8, 12, 24, 36, and 48 h after injection. B) H22 tumor‐bearing mice's ex vivo fluorescence images of dissected organs of the mice 48 h post‐injection. C)Schematic diagram of drug administration. D) Tumor growth curves of different groups in the whole experiment period. E) Weight of these harvested tumor tissues. F) Representative photographs of tumors excised from each treatment group on day 15. G) Immunohistochemical staining of tumor tissues derived from H22‐bearing tumor mice. H&E staining and immunohistochemistry staining of CD31, E‐cad, MMP‐9. H) The expression of apoptosis pathway‐relative proteins (cleaved caspase 9, cleaved caspase 8, cleaved caspase 3, Bcl‐2, and BAX) evaluated by western blot. I) quantitative evaluation of the expression level of these proteins. All experiments were repeated four times (n = 4), and data are presented as mean ± SD. One‐way ANOVA with Tukey's multiple comparison test was used for statistical analysis. *p < 0.05.

To evaluate the biodistribution of RBCm@R8‐Lip in the tumor tissue in vivo, DiR‐loaded liposomes were intravenously injected into H22 tumor‐bearing nude mice and monitored by an in vivo imaging system (IVIS) at scheduled time points. As shown in **Figure** **3**A, the fluorescence signals of DiR/RBCm@R8‐Lip were obviously detected in the tumor site at 1 h post‐injection and gradually increased with time, and maintained at a high level up to 48 h, which was significantly higher than that of free DiR, DiR/Lip and DiR/R8‐Lip. Such evidence suggested that the long circulation effect of RBC membrane camouflage strategy and targeting effect of R8 peptide could help RBCm@R8‐Lip efficiently deliver into H22 tumor sites. After 48 h post‐treatment, the mice were sacrificed, and the major organs and tumor tissues were excised for ex vivo imaging. In Figure 3B, the fluorescence intensity of the DiR/RBCm@R8‐Lip around the tumor was much higher than that of the DiR/@R8‐Lip, DiR/Lip, Free DiR groups. These results indicated that the in vivo extended circulation and tumor‐targeted accumulation can be efficiently enhanced by encapsulation within RBCm@R8‐Lip. Next, we evaluated the antitumor effects of biomimetic liposomes. The mice were randomly divided into five groups, and intravenously administered with saline, TP+Cel, Cel+TP/Lip, Cel+TP/R8‐Lip, and Cel+TP/RBCm@R8‐Lip with TP dose of 0.024 mg kg^−1^ and Cel dose of 3 mg kg^−1^ every two days for a total of seven treatments on H22 tumor‐bearing mice (Figure 3C). During the treatment period, the tumor growth curves were measured. As shown in Figure 3D, Cel+TP/RBCm@R8‐Lip administration significantly slowed down the growth rate of the tumor, suggesting dramatically improved therapeutic efficacy against the tumor. Moreover, the tumor weight (Figure 3E) showed a similar trend to the tumor volume (Figure 3F) in each group, biomimetic liposome group also showed minimum tumor weight compared to other groups. These findings indicate that biomimetic liposomes also have better anti‐tumor effects in vivo.

Meanwhile, the effect of biomimetic liposomes in migratory metastasis and apoptosis‐related pathways was further analyzed. As shown in Figure 3G, severe apoptotic damage was found in the H22 tumors of the Cel+TP/RBCm@R8‐Lip treated group compared with other groups, which was consistent with the abovementioned animal experiment results. These findings were also evaluated by immunohistochemistry for CD31, E‐cad, and MMP‐9. It is well known that CD31 is one of the vascular endothelial cell markers that can be used to assess tumor angiogenesis, and the expression level of E‐cad and MMP‐9 can reflect tumor cell metastasis.^[^ ^21^ ^]^ We found that the expression levels of CD31 and MMP‐9 were significantly reduced in the Cel+TP/RBCm@R8‐Lip group compared with other groups, while the number of E‐cad positive cells was higher than that of the other groups, indicating the excellent antiangiogenic ability of biomimetic liposome. WB analysis was also employed to evaluate the levels of apoptosis pathway proteins such as cleaved caspase 9, cleaved caspase 8, cleaved caspase 3, Bcl‐2, and Bax. As shown in Figure 3H,I, compared with the control group, the protein levels of cleaved caspase 9, cleaved caspase 8, cleaved caspase 3, and BAX were remarkably elevated by Cel+TP/RBCm@R8‐Lip, and fewer Bcl‐2 expressions appeared in biomimetic liposome group.

### 2.6 In Vivo Biodistribution and Antitumor Efficacy Against 4T1 Tumor‐Bearing Mice

**Figure 4:**
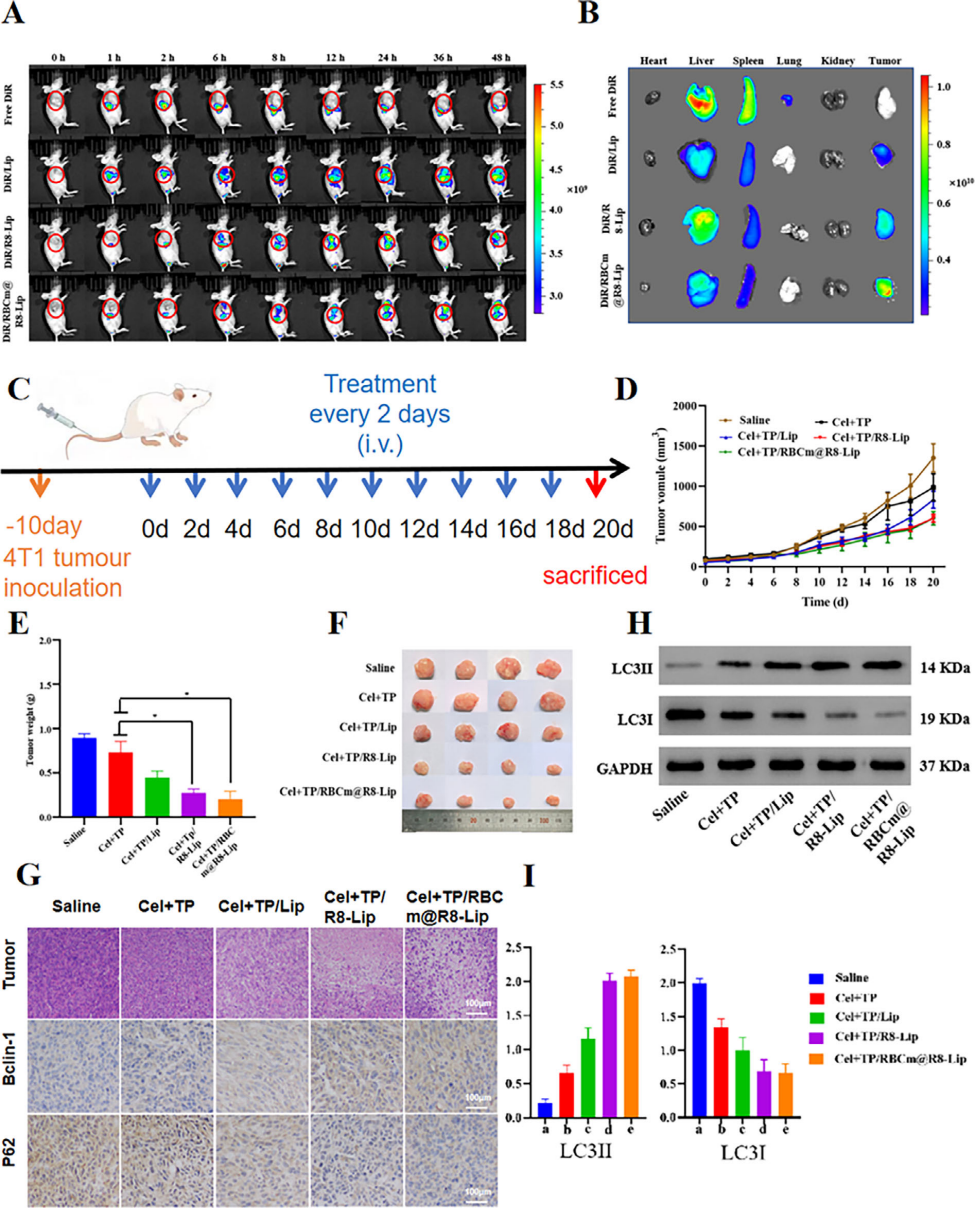
In vivo biodistribution and anti‐tumor effects of biomimetic liposomes in 4T1‐bearing tumor mice model. A) 4T1 tumor‐bearing mice's fluorescent distribution in vivo after vein injection at predetermined time intervals, i.e., 0, 1, 2, 6, 8, 12, 24, 36, and 48 h after injection. B) 4T1 tumor‐bearing mice's ex vivo fluorescence images of dissected organs of the mice 48 h post‐injection. C)Schematic diagram of drug administration. D) Tumor growth curves of different groups in the whole experiment period. E) Weight of these harvested tumor tissues. F) Representative photographs of tumors excised from each treatment group on day 20. G) Immunohistochemical staining of tumor tissues derived from 4T1‐bearing tumor mice. H&E staining and immunohistochemistry staining of Bclin‐1, P62. H) The expression of autophagy pathway‐relative proteins (LC3II, LC3I) evaluated by western blot. I) quantitative evaluation of the expression level of these proteins. All experiments were repeated four times (n = 4), and data are presented as mean ± SD. One‐way ANOVA with Tukey's multiple comparison test was used for statistical analysis. ^*^ *p* < 0.05.

To investigate the versatility of biomimetic liposomes in in vivo anti‐tumor therapy, 4T1 tumor bearing mice model was further established. We first investigated the in vivo fate of liposomes in 4T1 tumor bearing mice. Similarly, DiR/RBCm@R8‐Lip also showed the strongest fluorescence signals in tumor site compared with free DiR, DiR/Lip, and DiR/R8‐Lip (**Figure** **4**A). In ex vivo imaging of main organs after 48 h post‐treatment, DiR/RBCm@R8‐Lip exhibited much higher accumulation in the tumor site, owing to the RBC membrane shelter and R8 mediated endocytosis (Figure 4B). Next, we evaluated the antitumor effects of biomimetic liposomes against 4T1 tumor bearing mice. The mice were randomly divided into five groups, and intravenously administered with saline, TP+Cel, Cel+TP/Lip, Cel+TP/R8‐Lip, and Cel+TP/RBCm@R8‐Lip with equal doses of drugs every two days for a total of ten treatments (Figure 4C). As shown in Figure 4D, Cel+TP/RBCm@R8‐Lip treated group showed enhanced tumor inhibition effect. It was found that there was little difference in tumor size between the Cel+TP/RBCm@R8‐Lip group and the Cel+TP/R8‐Lip group, but the tumor weight was significantly reduced in the Cel+TP/RBCm@R8‐Lip group, as shown in Figure 4E,F. These findings indicated biomimetic liposomes also have better anti‐tumor effects 4T1 tumor model.

Furthermore, immunohistochemistry and WB analysis were also employed to evaluate the levels of autophagy pathway. As displayed in Figure 4G, the levels of Bclin‐1 and P62 indicated that Cel+TP/RBCm@R8‐Lip could exert antitumor effects by promoting autophagy of tumor cells. In Figure 4H biomimetic liposome remarkably elevated the protein levels of LC3II, and lowered LC3I expressions. The results indicated that biomimetic liposomes could exert antitumor effects by acting on autophagy‐related pathways.

### 2.7 Biocompatibility

To ensure the biosafety of drug formulations for further intravenous injection, the hemolysis analysis of R8‐Lip and RBC@R8‐Lip was conducted against red blood cells. PBS and water were used as the positive and negative control, respectively. As shown in Figure S2B (Supporting Information), the R8‐Lip exhibited slight hemolysis likely due to the cation property. In contrast, the RBCm@R8‐Lip exhibited nearly negligible (less than 3%) hemolytic activity at concentrations of up to 50 µg/mL did not induce erythrocytes to release hemoglobin, and no agglutination was visualized during incubation with RBC, implying that RBC membrane camouflage strategy reduced non‐specific interactions with the RBC membrane, resulting in good biocompatibility for intravenous administration.

Considering the severe toxicity of TP and Cel, we assess the toxic effect of biomimetic liposome by H&E and serum biochemistry analysis in both H22 and 4T1 models. As shown in Figures S3 and S4 (Supporting Information), the biomimetic liposome group had less severe lesions. Besides, ALT, AST, CREA, UA, and UREA levels in blood were significantly higher in the Cel+TP and liposome groups Instead of the biomimetic liposome group.

## 3 Conclusion

In summary, we successfully developed an RBC membrane‐dressed and cell‐penetrating peptide‐modified biomimetic liposome. The obtained Cel+TP/RBCm@R8‐Lip displayed significantly high drug encapsulation and excellent stability. Attributed by camouflage of RBC membrane and R8‐mediated tumor cell internalization, the biomimetic liposome has been demonstrated to show excellent antitumor effects both in breast cancer and liver cancer models. Immunohistochemical and WB analysis further illustrated that Cel+TP/RBCm@R8‐Lip achieved anticancer effects that were associated with the pathways of tumor cell migration, autophagy, and apoptosis. Reminiscent of natural chemotaxis, this work provides a facile dual‐modification strategy for targeted drug delivery, offering new solutions to the clinical application of CPPs‐based drugs.

## 4 Experimental Section

### Synergistic Effect of TP and Cel

The cytotoxicity of the TP/Cel mixture with increasing concentrations of TP and Cel but constant molar ratio (TP/Cel = 1/100) were studied by CCK‐8 assay for HepG2 (human liver cancer cell line) and MCF‐7 (human breast cancer cell line). Briefly, cells were treated with different concentrations (7.5, 10, 15, 20, 25, and 30 nm) of TP, Cel and the molar ratio of TP and Cel was 1:100 for 48 h. Then, the CCK‐8 solution was added to each well were incubated with cells for 4 h. OD of the formazan product was measured at 450 nm using a microplate reader (Thermo Fisher Scientific, Inc.). GraphPad software was used to calculate the half inhibitory concentration 50 (IC_50_).^[^ ^22^ ^]^

### Preparation of RBC Vesicles

RBC vesicles were prepared following the previously reported method with a few modifications.^[^ ^23^ ^]^ Fresh whole blood was collected from the mice and stored in heparin tubes for anticoagulation. Then, the blood was centrifuged at 3000 rpm for 5 min at 4 °C to remove the plasma. After washing the collected RBCs with phosphate‐buffered saline (PBS) 3 times, the resultant RBCs were via a hypotonic treatment by redispersing washed RBCs into the 0.25×PBS for 1 h at 4 °C. Subsequently, the RBCs solution was centrifuged at 12 000 rpm for 5 min at 4 °C until the supernatant appeared colorless. Finally, the RBC ghosts were obtained and placed in the −80 °C refrigerator for storage. Then, the RBCv (Red blood cell membrane vesicle) was prepared from the obtained RBC ghosts by sonic extrusions. Briefly, the RBC ghosts were resuspended in water with sonication for 3 min at a frequency of 40 kHz and a power of 100 W. The resultant RBCv was stored in water at 4 °C before use.

### Preparation and Characterization of Cel+TP/RBCm@R8‐Lip

Cel/TP‐loaded core liposomes were primarily made by regular ethanol injection method.^[^ ^24^ ^]^ Specifically, the stock solution of TP and Cel was prepared at concentrations of 1 and 10 mg mL^−1^, the mixture of egg yolk lecithin, cholesterol and dspe‐peg 2000‐mal at a mass ratio of 10.5:2.625:3 was dissolved in 335 µL ethanol. Respectively. And mixed with 65 µL TP solution and 600 µL Cel solution. Then, dropwise addition of the above mixture into 9 mL deionized water was implemented with continuous stirring at 60 °C for 1 h. The liposome suspension was sonicated for 5 min at 50% amplitude in an ice bath using an ultrasonic cell disruption system. and then passed through a 0.45 µm millipore filter. The liposome suspension was stored at 4 °C. For preparing Cel+TP/R8‐Lip, the stock solution of R8 was prepared at concentrations of 1 mg mL^−1^, 500 µL R8 solution was mixed with the above liposome suspension and incubated at ambient temperature for 1 h. Cel+TP/R8‐Lip were obtained after filtration with a 0.45 µm syringe filter. To encapsulate Cel+TP/R8‐Lip into RBC vesicles, 1 mL RBC vesicles were mixed with 1 mL Cel+TP/R8‐Lip, the mixture was sonicated for 3 min (40 kHz, 100 W) and passed through 0.45 µm filter to remove excess RBC vesicles.^[^ ^25^ ^]^ The Cel+TP/RBCm@R8‐Lip were obtained were stored at 4 °C for further experimentation.

### Protein Characterization

Sodium dodecyl sulfate‐polyacrylamide gel electrophoresis (SDS‐PAGE) was used for qualitative identification of protein on the membrane surface.^[^ ^26^, ^27^ ^]^ Specifically, the RBC vesicles, Lip, and RBCm@Lip were diluted with loading buffer as measured by the BCA assay kit and boiled in water for 5 min. Sample of 10 µL was loaded into the gel groove. Next, electrophoresis was carried out at a constant voltage of 90 V for 15 min and 110 V for 90 min. The gel was stained with Coomassie blue, washed with deionized water, and then imaged. Furthermore, the CD47 protein derived from erythrocytes was determined by western‐blotting analysis (WB). All proteins in each sample were separated by 10% SDS‐PAGE and transferred onto polyvinylidene difluoride membranes (PVDF). The transferred PVDF membranes were blocked with 5% milk, and then incubated with primary antibodies against CD47 for 12 h at 4 °C. Finally, the membranes were incubated with HRP‐conjugated secondary antibodies and observed by the imaging system. To examine the in vitro immune activation of the biomimetic liposome carrier, liposome–protein complexes were prepared by incubating liposomes with plasma (3:1 v/v) at 37 °C for 30 min, which according to previous investigations with a little adjustment.^[^ ^28^ ^]^ Liposome–protein complexes were collected by centrifugation for 15 min at 14 000 rpm with PBS washing three times to remove unbound proteins. Next, protein contents of liposomes and liposome–protein complexes were measured with a BCA kit, the composition of the protein crown (PC) was investigated by SDS‐PAGE. Furthermore, the composition of PC was carried out by an Easy‐nLC 1200 system coupled with an Orbitrap Eclipse Mass Spectrometer (Thermo Fisher Scientific, USA) with an ESI nanospray source. Specifically, the target gel block was cut after electrophoresis and placed in an EP tube to decolorize until transparent, the proteins were reduced and alkylated using DTT and IAA, separately. Then, trypsin was added overnight at 37 °C, lyophilized samples were re‐solubilized by 0.1% FA and detected by LC–MS/MS, searched by Maxquant (1.6.5.0), the false positive rate of protein identification was controlled to less than 1%.

### Endocytosis by Macrophages

To study the endocytosis of the liposomes and biomimetic liposomes carrier by macrophages in the immune system, cellular uptake of liposomes carrier by RAW264.7 macrophage cells was evaluated by confocal laser scanning microscopy (CLSM, TCS SP8 SR; Leica, Weztlar, Germany) and flow cytometry (FCM, BD FACSVerseTM, Franklin Lake, NJ, USA). RAW264.7 cells were seeded at a density of 1 × 10^5^ cells well^−1^ into confocal dishes, C_6_ (Coumarin 6)‐loaded liposomes were added to RAW264.7 cells with C_6_ final concentration of 100 ng mL^−1^ at 37 °C for 4 h. Then washed thoroughly with PBS, fixed with 4% polyformaldehyde for 10 min and then stained with Hoechst 33 342 for nuclei. Finally, the uptake situation was evaluated by CLSM imaging. RAW264.7 cells were seeded at a density of 3.5 × 10^5^ cells well^−1^ in 6‐well dishes. After being incubated overnight and treated with the above drugs for 4 h. Finally, the cells were collected and suspended in PBS. Intracellular C_6_ fluorescence was detected by FCM and analyzed with FlowJo software.

### Cellular Uptake

The tumor cellular uptake tests were also conducted in FCM and CLSM. Two kinds of tumor cells were chosen and incubated in 6‐well plates with a density of 3 × 10^5^ cells well^−1^ and cultured overnight, respectively. The subsequent procedure was the same as that described in endocytosis by macrophages.

### In Vitro Cytotoxicity

HepG2 cells and MCF‐7 cells were seeded in 96‐well plates with an average density of 5 × 10^3^ cells per well, after overnight culture, the cells were incubated with fresh culture medium containing Cel+TP, Cel+TP/Lip, Cel+TP/R8‐Lip and Cel+TP/RBCm@R8‐Lip for 24 h and 48 h. 10 µL of CCK8 was added and co‐incubated with the cells for 4 h at 37 °C. Finally, the optical density was measured at 450 nm with a microplate plate reader. To further visualize the cytotoxicity of each group, HepG2 cells, and MCF‐7 cells were incubated with four groups for 24 h, including Cel+TP, Cel+TP/Lip, Cel+TP/R8‐Lip, and Cel+TP/RBCm@R8‐Lip. TP and Cel were administered at the equivalent concentration of 0.01 and 1 µm in groups, separately. All cells were then washed in PBS, treated them with Calcein‐AM and PI as per the manufacturer's instructions, and observed them with a fluorescence microscope.

### Apoptosis

HepG2 cells were plated in 12 well‐plate and cultured overnight. HepG2 cells were treated with five groups for 24 h, including control, Cel+TP, Cel+TP/Lip, Cel+TP/R8‐Lip, and Cel+TP/RBCm@R8‐Lip. TP and Cel were added at the equivalent concentration of 0.01 and 1 µm in groups, respectively. Cells were collected and treated with eBioscience Annexin V‐FITC Apoptosis Detection Kit (BMS500FI‐300), and then detected with flow cytometry within 4 h.

### Animals

The animal experiments were conducted under the permission of the Animal Ethics Committee of Chengdu University of Traditional Chinese Medicine (Chengdu, China) (permission number SYXK2020‐124), all experiments were performed in accordance with relevant regulations.

### In Vivo Biodistribution

The liposomes carrier local accumulation was further evaluated by the IVIS fluorescence imaging system. H22 and 4T1 tumor‐bearing nude mice were constructed by subcutaneous injection of ≈1 × 10^7^ H22 and 4T1 cells into the right flank of male ICR and female BALB/c nude mice, separately. Each tumor‐bearing mice were assigned randomly to four groups and injection of free DiR, DiR Lip, DiR/R8‐Lip, and DiR/RBCm@R8‐Lip via the tail vein at a DiR concentration of 0.5 mg kg^−1^, respectively. At the predetermined time points (0, 1, 2, 6, 8, 12, 24, 36, and 48 h) after administration, the changes in fluorescence intensity in mice were observed by the IVIS fluorescence imaging system (PerkinElmer). At the endpoint, mice were sacrificed to obtain the intestine and tumor, and immediately imaged.

### In Vivo Therapeutic Study

The H22 tumor‐bearing mice received an i.v. injection of 200 µL of saline, Cel+TP, Cel+TP/Lip, Cel+TP/R8‐Lip, and Cel+TP/RBCm@R8‐Lip with TP dose of 0.024 mg kg^−1^ and Cel dose of 3 mg kg^−1^. Tumor size and body weight were monitored during administration. At the end of the experiment, their blood samples and major organs (including hearts, livers, spleens, lungs, and kidneys) were collected for further tests. To investigate the generalizability of this co‐delivery system for the treatment of cancer. 4T1 cells were used to establish another tumor‐bearing mouse model. The experimental method was consistent with that used for H22 tumor‐bearing mice. Furthermore, the effect of liposomes treatments on tumor aggressive metastasis and angiogenesis suppression efficacy was evaluated by immunohistochemistry staining of CD31, E‐cad, MMP‐9, Bclin‐1and P62. In addition to that, western blotting (WB) was also performed to assess the expression of signaling proteins involved in apoptosis and autophagy, including cleaved caspase 9, cleaved caspase 8, cleaved caspase 3, Bcl‐2, BAX, LC3II, and LC3I.

### Biosafety

Membrane disruption of erythrocytes was used to assess the hemolytic effect of liposomes carrier.^[^ ^29^ ^]^ 2% erythrocytes were mixed with various concentrations (1, 10, 20, 30, 50, and 100 µg mL^−1^) of liposomes carrier solution in equal volume. Similarly, 2% erythrocytes were mixed with pure water was used as a positive control, and mixed with physiological saline was used as a negative control. The mixture was incubated at 37 °C for 4 h. Afterward, all samples were centrifuged at 3000 rpm for 5 min at 4 °C before measuring the absorbance of the supernatant at 540 nm. The percentage of hemolysis was calculated according to the following formula: Hemolysis (%) = (I*_sample_* – I*_neg_* / I*_pro –_* I*_neg_*) ×100%. Finally, the histological images of the main organs were evaluated using H&E staining and determined several blood biochemical indexes including ALT, AST, CREA, UA, and UREA.

### Statistical Analysis

Statistical evaluations were conducted utilizing GraphPad Prism version 8.0.2 software. All data were expressed as the means ± standard deviation (SD). Statistical difference between experimental results was assessed by one‐way ANOVA. Statistical significance was set at *p* < 0.05.

## Conflict of Interest

The authors declare no conflict of interest.

## Supporting information



Supporting Information

## Data Availability

The data that support the findings of this study are available in the supplementary material of this article.
