# Supplementary material for: RBC Membrane‐Camouflaged Nanosystem‐Mediated Synergistic Drug Combination for Enhanced Anti‐Tumor Therapy
Source: Adv Healthc Mater. 2025 May 7;14(26):2500446. doi: 10.1002/adhm.202500446 (PMC12506840; doi:10.1002/adhm.202500446)
Supplement: Supplementary file 1 — Supporting Information [file ADHM-14-0-s001.docx]

Supporting Information

**RBC Membrane-Camouflaged Nanosystem-mediated Synergistic Drug Combination for Enhanced Anti-tumor Therapy**

Qian Cheng^1^, Xuemei Zhong^1^, Lu Deng^1^, Xinling He^1^, Miaoxizi Luo^1^, Ruibing Wang^2^* and Jinming Zhang^1^*

1. Q. Cheng, X. Zhong, L. Deng, X. He, M. Luo and J. Zhang

State Key Laboratory of Southwestern Chinese Medicine Resources, Pharmacy School, Chengdu University of Traditional Chinese Medicine,

Chengdu, 611130, China

E-mail: cdutcmzjm@126.com

1. R. Wang

State Key Laboratory of Quality Research in Chinese Medicine, Institute of Chinese

Medical Sciences, University of Macau,

Taipa, Macau SAR 999078, China.

E-mail: rwang@um.edu.mo

**Key words:** RBC membrane-camouflaged, nanosystem, CPPs, drug combination, anti-tumor

*Materials:* Triptolide and Celastrol of 98% purity were obtained from Desite Biological Technology Co., Ltd. (Chengdu, China). Egg Yolk Lecithin was purchased from Aiweituo Pharmaceutical Technology Co., Ltd. (Shanghai, China). Cholesterol was procured from Aladdin Biochemical Technology Co., Ltd. (Shanghai, China). depe-peg2000-mal was provided by Ruixi Biotechnology Co., Ltd. (Xi'an, China). Cell penetrating peptide R8 was procured from Qiangyao Biotechnology Co., Ltd. (Shanghai, China). Coumarin-6 (C6) was provided by Yuanye Co., Ltd. (Shanghai, China). 1,1′-Dioctadecyltetramethyl indotricarbocyanine iodide (DiR) was obtained from Mei Biotechnology Co., Ltd. (Dalian, China). Hoechst 33342 and Fluorescent Mounting Media were provided by BD Biosciences Co., Ltd. (NJ, USA). Cell Counting Kit was supplied by Seville Biotechnology Co., Ltd. (Wuhan, China). CD47 Rabbit Polyclonal Antibody was supplied by Shanghai Biyuntian Biotechnology Co., Ltd. (Shanghai, China). Blood collection vessel containing heparin sodium was purchased from Haifuda Technology Co., Ltd. (Beijing, China). The human liver cancer HepG2 cell line, human breast cancer MCF-7 cell line, murine liver cancer H22 cell line, murine breast cancer 4T1 cell line and Leukemic monocyte macrophage cell lines (RAW264.7 macrophage) were purchased from the American Type Culture Collection (ATCC) (Manassas, VA, USA). All in vivo experiments were carried out under the guidelines approved by the Institutional Animal Care and Use Committee (IACUC) of Chengdu University of TCM. Male SD rats (250~300 g), male ICR mice (18~20 g), male nude mice (16~18 g), female BALB/c mice (16~18 g) and femeal nude mice (16~18 g) were provided by SPF (Beijing) Biotechnology Co., Ltd. (Beijing, China).


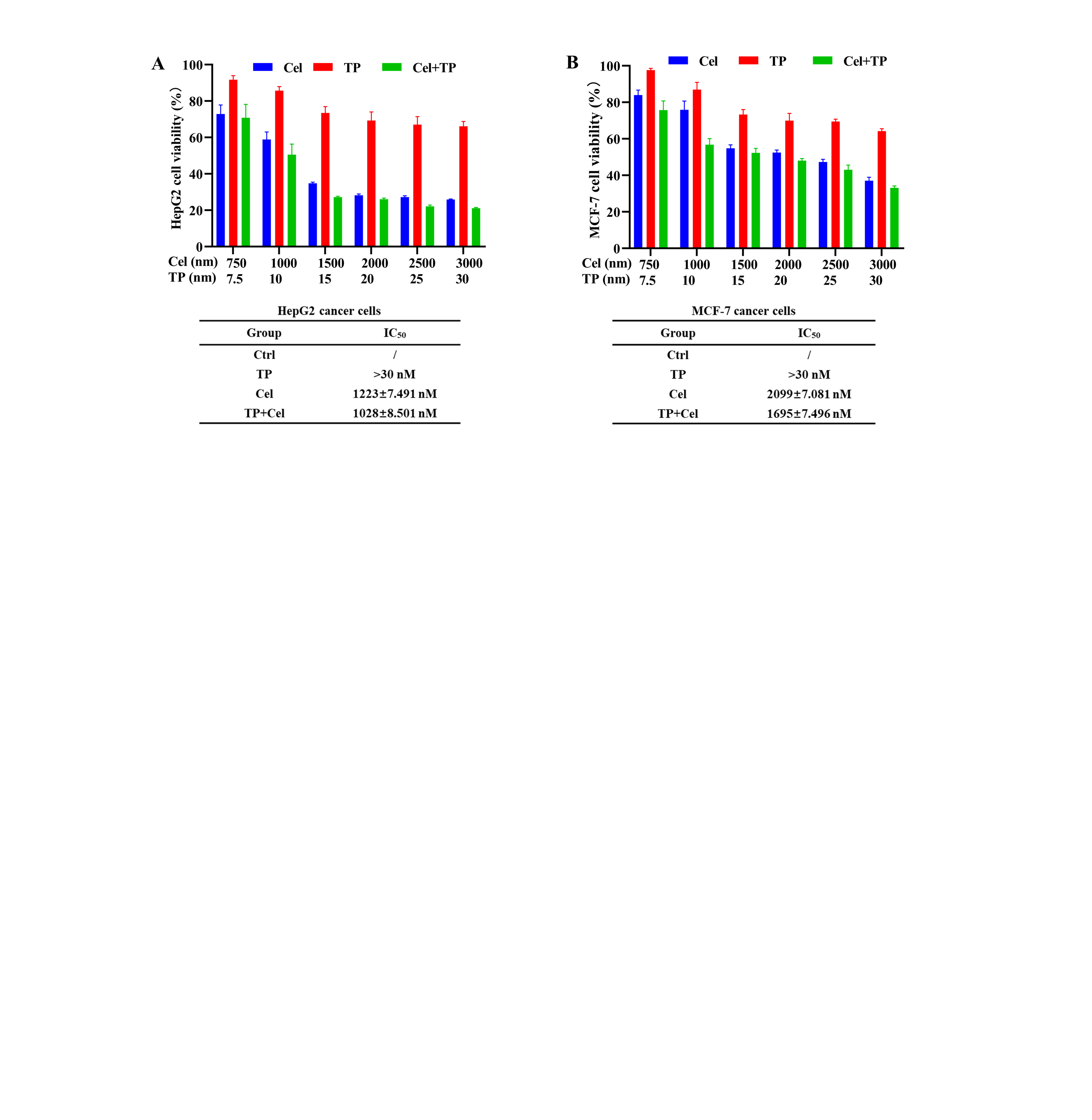


**Figure S1.** Cytotoxicity and IC50 values of Cel, TP, Cel+TP against HepG2 (A) and MCF-7 (B) cells with 24 h treatment in different culture media.


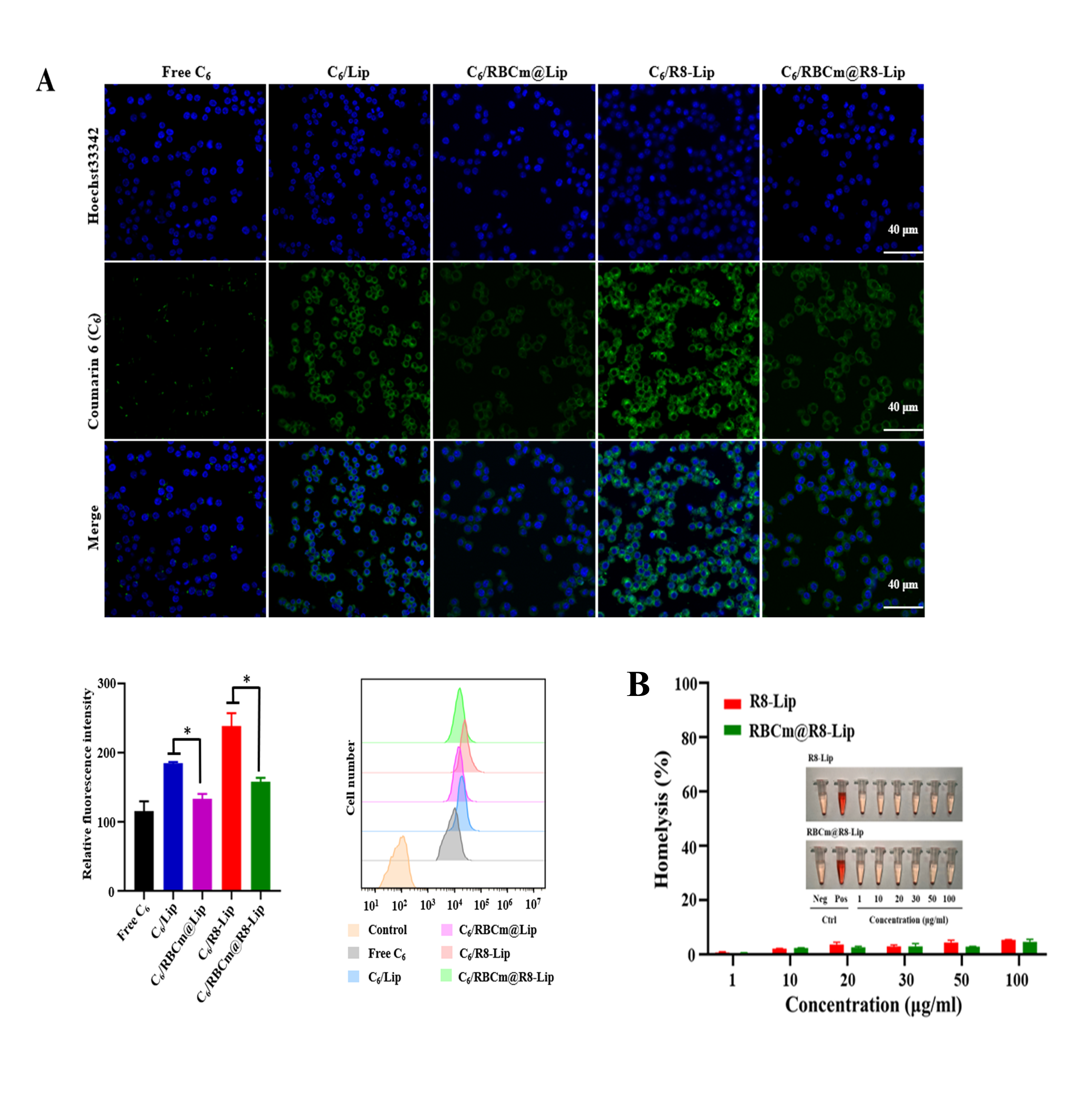


**Figure S2.** (A) Cellular uptake (including quantitative analysis) of Free C6, C6-loaded liposomes and C6-loaded biomimetic liposomes by RAW264.7 cells. Scale bar: 40 μm. (B) Hemolysis test.


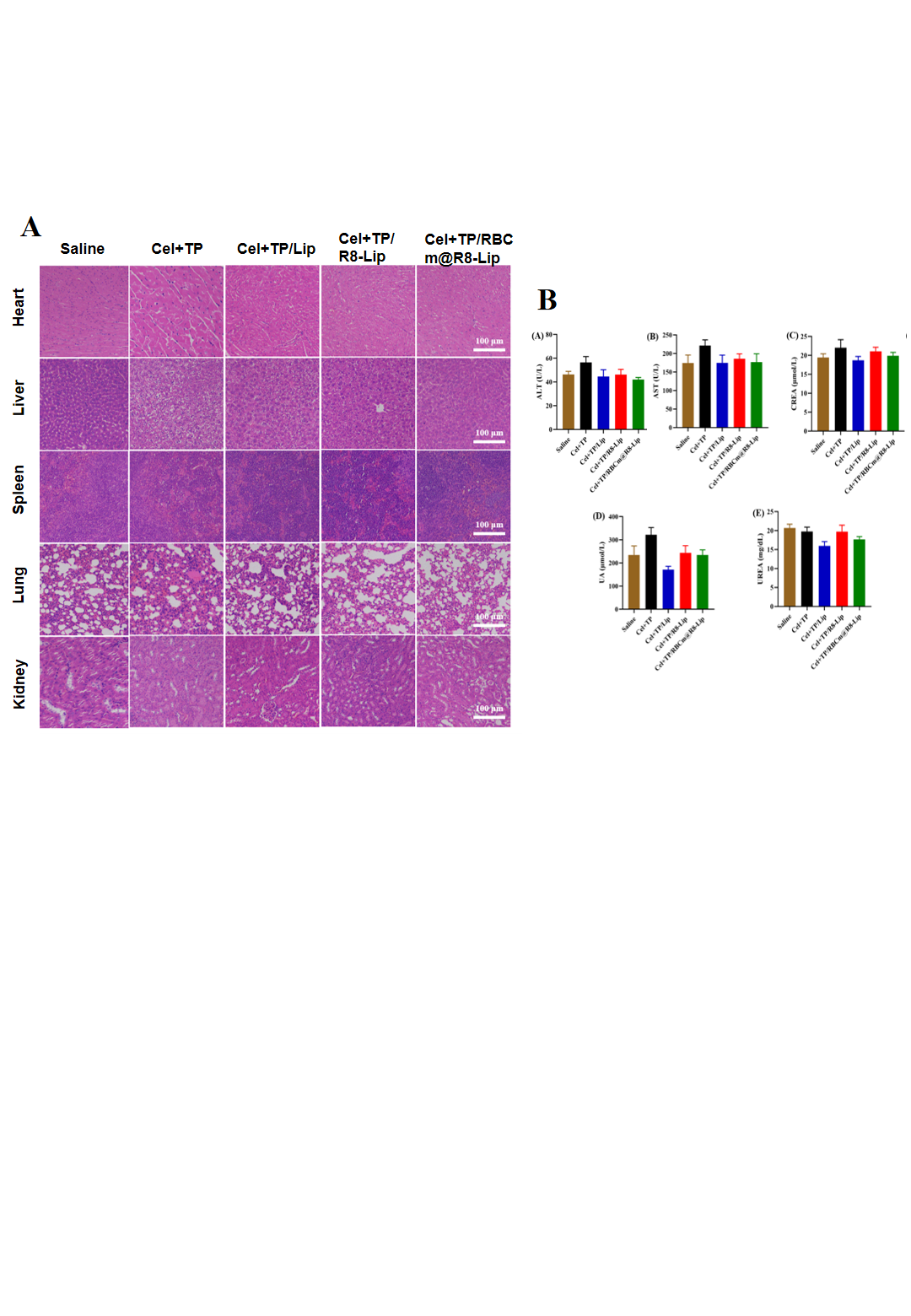


**Figure S3.** (A) H&E histological analyses of main organs after various treatments of the H22 tumor model. Scale bar: 100 μm. (B)The content of ALT, AST, CREA, UA and UREA in blood after treatment.


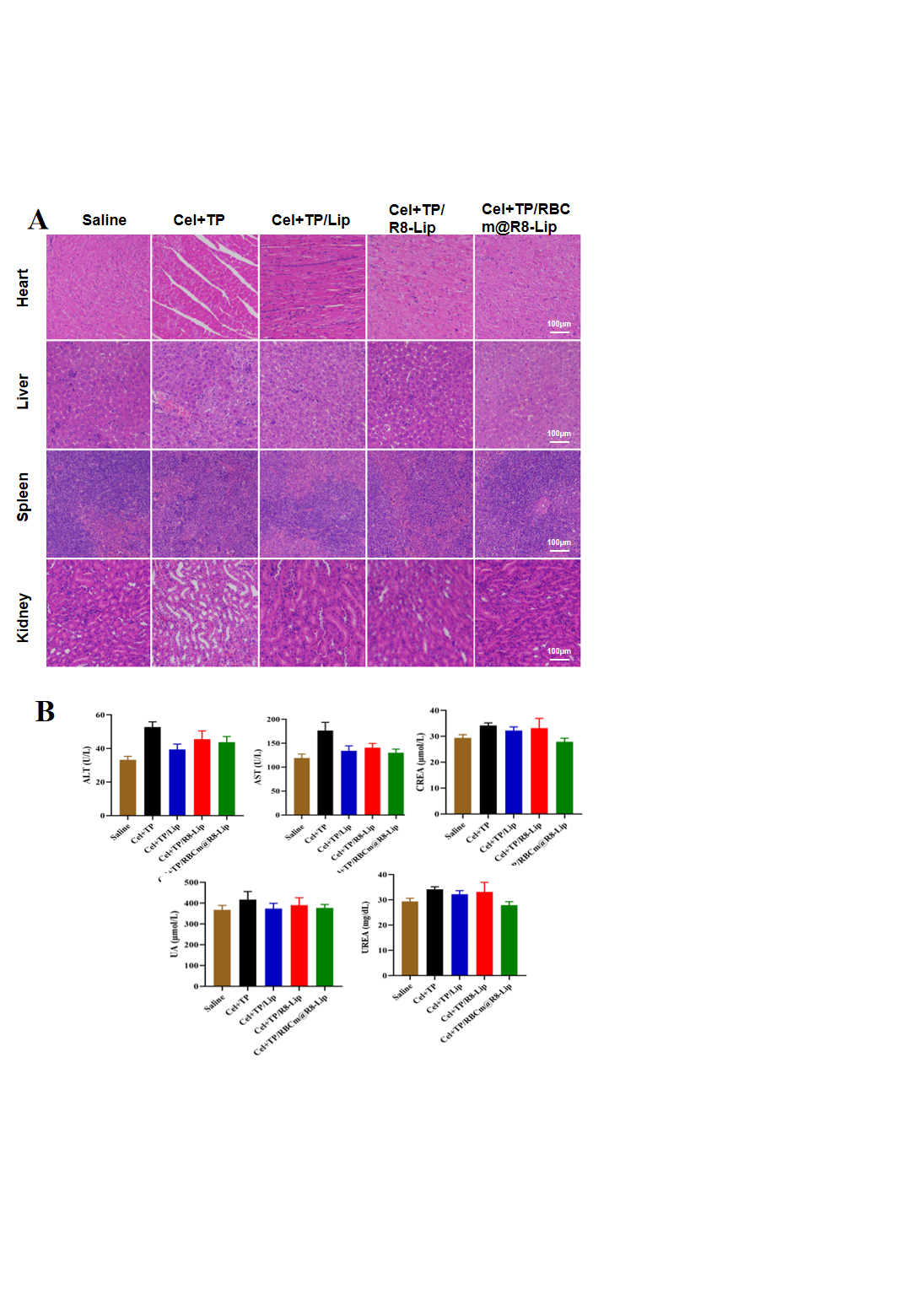


**Figure S4.** (A) H&E histological analyses of main organs after various treatments of the 4T1 tumor model. Scale bar: 100 μm. (B)The content of ALT, AST, CREA, UA and UREA in blood after treatment.


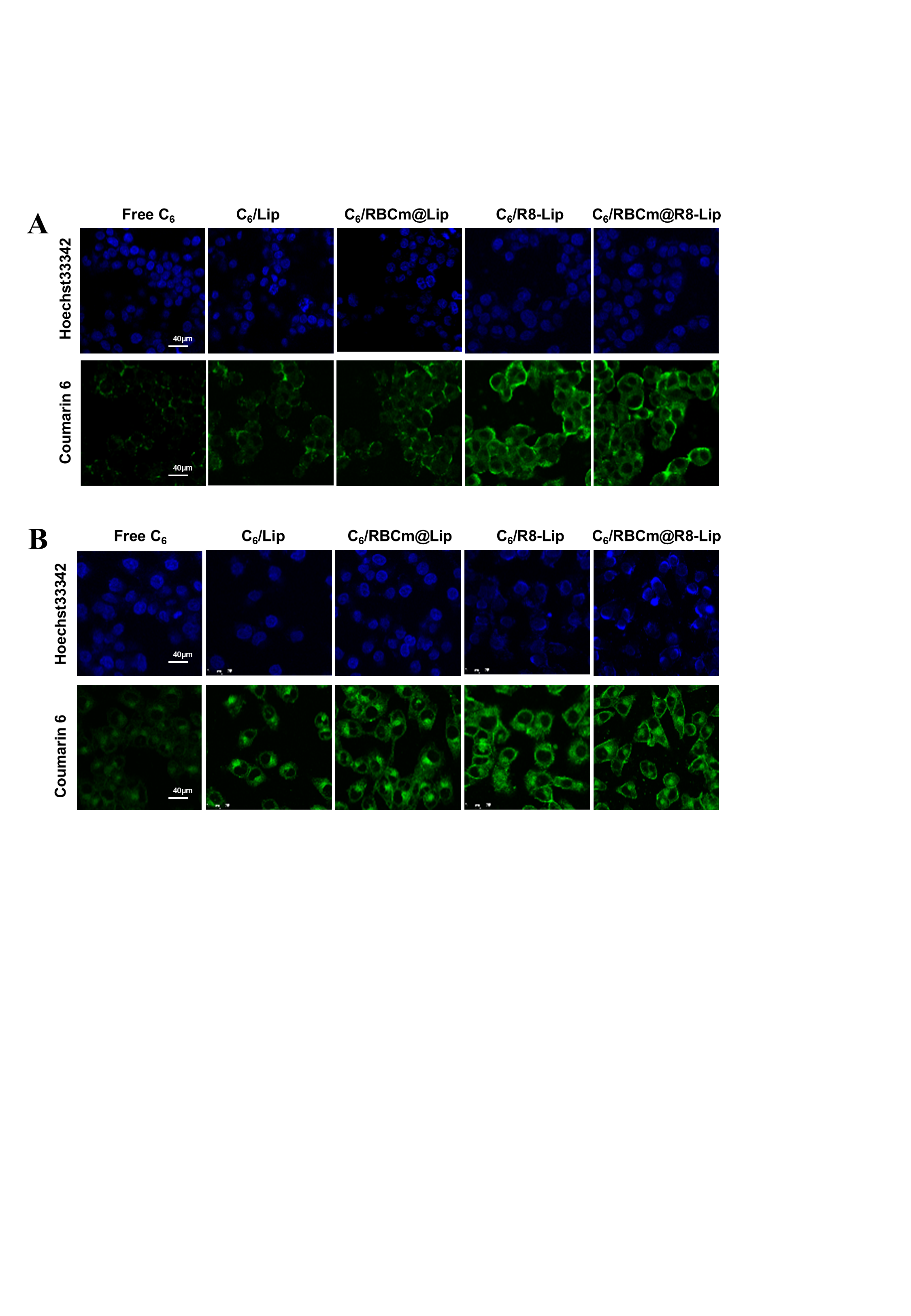


**Figure S5.** Representative cellular uptake images of HepG2(A) and MCF-7(B) cells after incubation with Free C6, C6/Lip, C6/RBCm@Lip, C6/R8-Lip, C6/RBCm@R8-Lip, respectively, for 4 h by CLSM observation and by FCM. Scale bar: 40 μm.


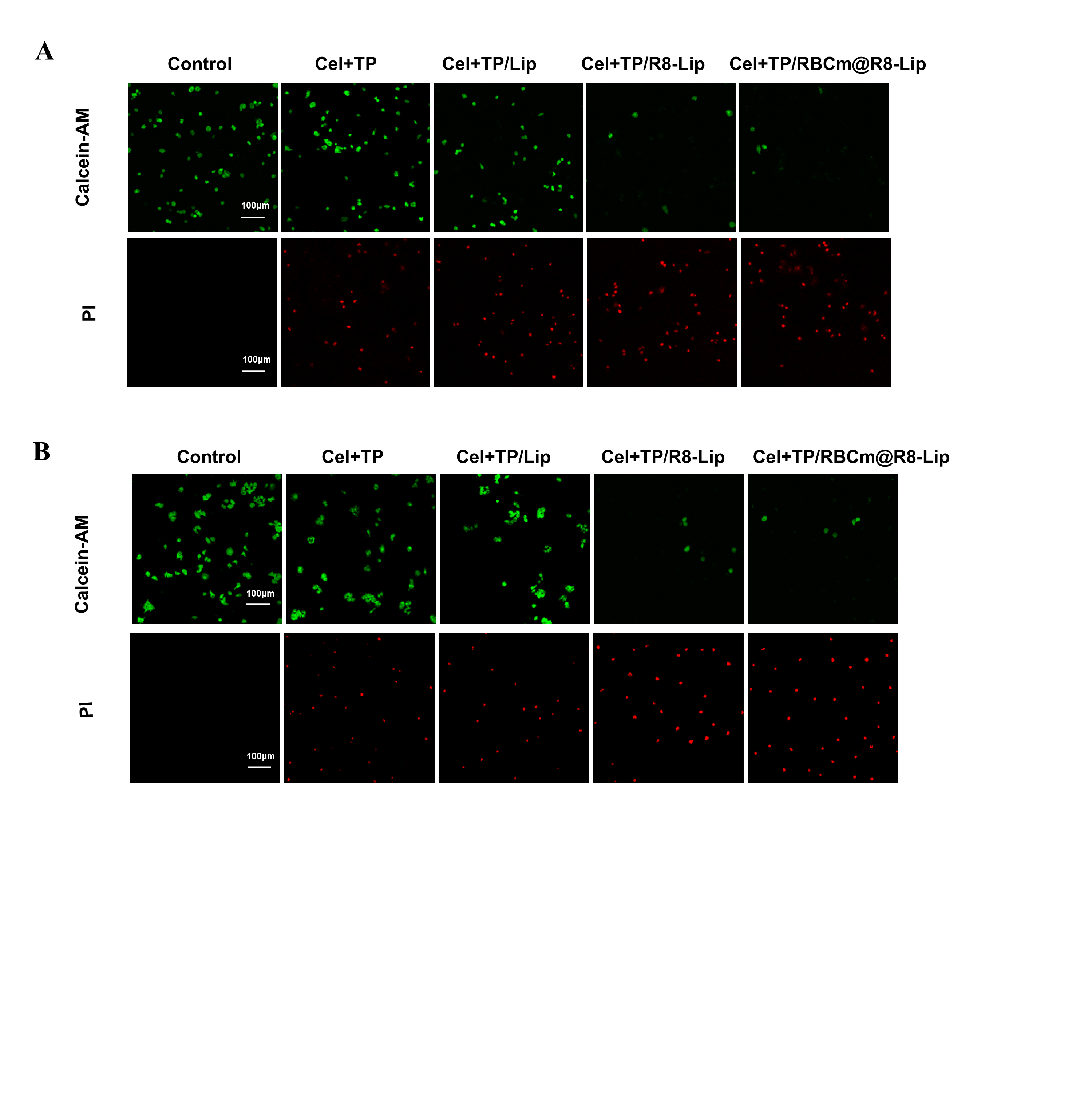


**Figure S6.** Detection of live/dead cells after various therapies of HepG2(A) and MCF-7(B). Scale bar: 100 μm.
